# Circulating Exosomal CircMYC Is Associated with Recurrence and Bortezomib Resistance in Patients with Multiple Myeloma

**DOI:** 10.4274/tjh.galenos.2020.2020.0243

**Published:** 2020-11-19

**Authors:** Yanwei Luo, Rong Gui

**Affiliations:** 1 The Third Xiangya Hospital of Central South University, Department of Blood Transfusion, Changsha, China

**Keywords:** Circular RNA, Biomarker, Drug resistance, Overall survival, Hematological tumor

## Abstract

**Objective:**

Studies have shown that serum circRNA can be used as a biomarker for many tumors. However, the role of exosomal circRNA in prognostic evaluation in patients with multiple myeloma (MM) remains unclear. In this study, we aimed to analyze the role of circulating exosomal circMYC in the relapse and prognosis of patients with MM.

**Materials and Methods:**

Circulating exosomes from 122 patients with MM and 54 healthy people were isolated. Quantitative polymerase chain reaction was performed to measure circMYC exosomal expression. Kaplan-Meier survival curves with log-rank testing were used for estimating significance in survival rates. A Cox regression model was used for univariate and multivariate analysis.

**Results:**

Compared with healthy people, the expression level of serum exosomal circMYC was significantly increased in patients with MM. In addition, the expression of circMYC in circulating exosomes in bortezomib-resistant patients was significantly higher than that in non-resistant patients. The expression level of exosomal circMYC was correlated with deletion 17p, t(4;14), Durie-Salmon staging, and the International Staging System. Univariate and multivariate Cox regression analysis found that a high exosomal circMYC level was an independent predictor of poor prognosis in patients with MM. The patients with high exosome circMYC expression had higher relapse rates and higher mortality rates. The overall survival rate and progression-free survival rate of MM patients with high exosomal circMYC expression were lower than those of patients with low exosomal circMYC expression.

**Conclusion:**

These findings suggest that circulating exosomal circMYC has great potential as a biomarker for the diagnosis and prognosis of MM.

## Abstract (tr)

**Amaç:**

Çalışmalar göstermiştir ki serum circRNA pekçok tümör için biyobelirteç olarak kullanılabilir. Ancak, multiple myelomlu (MM) hastalarda eksozomal circRNA’nın rolü belirsizdir. Bu çalışmada dolaşan eksozomal circMYC’nin MM’li hastaların nüks ve prognozundaki rolünü analiz etmeyi hedefledik.

**Gereç ve Yöntemler:**

MM’li 122 hasta ve 54 sağlıklı kişiden dolaşan eksozomlar izole edildi. Kantitatif polimeraz reaksiyonu circMYC eksozomal ekspresyonunu ölçmek için uygulandı. Sağkalım oranının önemini tahmin etmek için log-rank testiyle beraber Kaplan-Meier sağkalım eğrileri kullanıldı. Tek değişkenli ve çok değişkenli analiz için Cox regresyon modeli kullanıldı.

**Bulgular:**

Sağlıklı kişilerle karşılaştırıldığında MM’li hastalarda serum eksozomal circMYC ekspresyon düzeyleri anlamlı ölçüde artmıştı. İlaveten, dolaşan eksozomlarda circMYC ekspresyonu bortezomibe dirençli hastalarda dirençli olmayanlara göre anlamlı ölçüde yüksekti. Eksozomal circMYC ekspresyon düzeyi delesyon 17p, t(4;14), Durie-Salmon evrelemesi ve Uluslararası Evreleme Sistemi ile bağlantılıydı. Tek değişkenli ve çok değişkenli Cox regresyon analizi yüksek eksozomal circMYC düzeyinin MM’li hastalarda kötü prognozun bağımsız göstergesi olduğunu gösterdi. Yüksek eksozom circMYC ekspresyonu olan hastalarda daha yüksek nüks ve mortalite oranı vardı. Yüksek eksozomal circMYC ekspresyonu olan MM’lu hastaların genel sağkalım ve ilerlemesiz sağkalım oranı düşük eksozomal circMYC ekspresyonu olan hastalardan daha düşüktü.

**Sonuç:**

Bu bulgular dolaşan eksozomal circMYC’nin MM tanı ve prognozu için biyobelirteç olma potansiyeli olduğunu düşündürmektedir.

## Introduction

Multiple myeloma (MM) is the most common type of human plasma cell disease, accounting for about 10% of all hematological tumors [1]. In patients with MM, the malignant plasma cells in the bone marrow rapidly proliferate and secrete a large number of monoclonal immunoglobulins in different parts of the body, which causes a series of clinical manifestations such as renal insufficiency, bone destruction, anemia, and hypercalcemia [2]. Although in the past 10 years, with the continuous development of new drugs and the development of stem cell transplantation, the overall survival time and quality of life of patients with MM have been greatly improved, MM is still an incurable disease [3]. Bortezomib (BTZ) is one of the most successful chemotherapeutics for MM [4]. However, although BTZ has achieved clinical success, some patients have failed BTZ treatment due to primary or secondary drug resistance [5]. Therefore, finding effective markers for prognosis and new therapeutic targets for MM has important clinical significance. New characteristic target molecules may provide the possibility of finding new methods for the treatment and prognostic evaluation of MM.

Extracellular vesicles, including exosomes, have recently been identified as the third mediators of cellular communication [6]. Exosomes are a type of bilayer lipid membrane vesicle with a diameter of about 30 to 100 nm. They are found in almost all body fluids, including plasma, serum, saliva, milk, cerebrospinal fluid, and urine [7], and they are particularly abundant in the microenvironment of tumors. Exosomes contain a variety of proteins and nucleic acid components, among which the RNA molecules include mRNA, miRNA, lncRNA, and circRNA [8]. Exosomes can carry these functional substances to transfer them between cells, mediate information exchange, and regulate a variety of physiological and pathological activities such as cell protein synthesis, proliferation, differentiation, and antiviral response [9].

CircRNA is non-coding RNA with a circular structure, forming a covalently closed continuous loop containing hundreds to thousands of base pairs, which is not degraded by exonuclease [10]. CircRNA is stable and widely present in the body, such as in cytoplasm, nuclei, serum exosomes, and saliva [11]. Studies have found that circRNA plays an important role in normal physiological processes and the occurrence and development of various diseases, and it can be used as a clinical diagnostic marker for various diseases [12]. The application of high-throughput sequencing technology has identified a large number of circRNAs and led to the establishment of circRNA databases such as CircBase and CircNet [13,14]. Previous studies have shown that serum circRNA can be used as a biomarker for many tumors [15]. The circRNA circMYC (hsa_circ_0085533) is derived from the *MYC* gene and contains an exon circRNA transcript located on chromosome 8. CircMYC has been reported to promote the proliferation of breast cancer cells [16] and human melanoma cells [17]. However, the expression of circulating exosomal circMYC in patients with MM and its role in prognostic evaluation remain unclear. This study focused on the expression of circMYC in the serum exosomes of patients with MM and its role in the evaluation of relapse and drug resistance in these patients.

## Materials and Methods

### Human Samples

From January 2014 to January 2019, we collected peripheral serum samples and clinical case data of 122 patients diagnosed with MM in our hospital. We also collected the clinical and pathological data of the patients, including age (ranging from 41 to 76 years old), gender (79 male and 43 female patients), disease classification, and clinical stage [Durie-Salmon staging (DSS) and International Staging System (ISS)]. The diagnosis of MM patients was based on the diagnostic criteria of the International Myeloma Working Group [18]. In addition, 54 peripheral serum samples from healthy individuals were collected as a control group. The healthy controls were included based on the following criteria: no previous history of malignant tumors, and normal liver and kidney functions. The study was approved by the Ethics Committee of The Third Xiangya Hospital, Central South University. All subjects signed the informed consent form.

### Exosome Isolation and Identification

The serum was thawed on ice and then centrifuged at 2000x g for 30 min at 4 °C to remove debris. The supernatant was filtered through a 0.22-µm filter. The filtered supernatant was then transferred to a 50-mL ultrafiltration tube. The exosomes were extracted using an exosome isolation kit (EXOTC50A-1, System Biosciences, Palo Alto, CA, USA). Briefly, 0.5 times the volume of the extraction reagent was added and mixed well with the filtered supernatant, and this mixture was incubated at 4 °C overnight. After that, the samples were centrifuged at 10000x g for 60 min at 4 °C. The supernatant was discarded, and the exosome pellets were resuspended in PBS. Samples of 10 µL were used for transmission electron microscopy observations, and 100 µL was used for RNA extraction and quantitative polymerase chain reaction (qPCR) analysis.

### Transmission Electron Microscopy to Observe Exosomes

The morphologies of the exosomes were observed by transmission electron microscopy as previously described [19]. Briefly, the exosomes were fixed with 4% glutaraldehyde solution and then stained with phosphotungstic acid (2%) for 30 s. Morphology was observed under a transmission electron microscope at an acceleration voltage of 80 kV.

### Ribonuclease R Treatment

Ribonuclease R (RNase R) digests almost all linear RNA molecules, but it is not easy to digest circular RNAs. RNase R can be used to digest linear RNA and identify circular RNAs [20]. RNase R (10 U; Geneseed, Guangzhou, China) was added to 2.5 µg of total RNA and mixed well for 30 min of incubation at 37 °C. The qPCR experiments were then performed as described below.

### Quantitative Polymerase Chain Reaction

The Plasma/Serum Exosome Purification and RNA Isolation Mini Kit (Cat No. 58300, Thorold, Canada) was used to extract RNA from exosomes according to the manufacturer’s instructions. SYBR Premix EX Taq II (TaKaRa, Mountain View, CA, USA) was used for real-time qPCR on the ABI 7500 Real-Time PCR System (SeqGen, Inc., Torrance, CA, USA). GAPDH was used as an internal control. Three replicates were designed for each sample. The primers used were as follows: circMYC, forward: CTCACAGCCCACTGGTCCTC, reverse: TCCAGCAGAAGGTGATCCAG; GAPDH, forward: GAAAGCCTGCCGGTGACTAA, reverse: TTCCCGTTCTCAGCCTTGAC. Data are presented as fold change relative to the mean of circMYC in healthy subjects.

### Statistical Analysis

We used SPSS 21.0 (IBM Corp., Armonk, NY, USA) for statistical analysis. The unpaired t-test method was used to analyze the differences of the expression levels of circMYC in circulating exosomes between patients with MM and healthy people. The chi-square test was used for analyzing the differences between clinical features and circMYC expression. Kaplan-Meier survival curves with log rank testing were used for estimating significance in survival rates. A Cox regression model was used for univariate and multivariate analysis. The significance level was defined as p<0.05.

## Results

### Clinical Characteristics of Patients with MM

The clinical characteristics of 122 patients with MM are shown in Table 1. The total group of patients comprised 79 men and 43 women; the median age was 62 years (41-76 years). Eighty-four cases (68.9%) were stage I or II according to the ISS, while 38 cases (31.1%) were stage III; for DSS stages, 87 cases (71.3%) were stage I or II, while 35 cases (28.7%) were stage III. Regarding the type of monoclonal immunoglobulin, 71 cases (58.2%) were of the IgG type, 32 cases (26.2%) were IgA type, 9 cases (7.4%) were IgD type, and 10 cases (8.2%) were IgM type. The median hemoglobin level was 7.3 (4.5-12.8) g/dL and blood calcium was 2.5 (1.8-4.73) mmol/L.

### CircMYC Expression in Circulating Exosomes in Patients with MM

**Figure 1:**
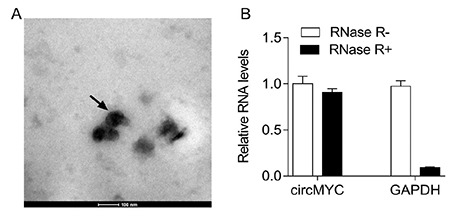
CircMYC expressed in exosomes from patients with multiple myeloma. (A) The morphology of circulating exosomes was observed by transmission electron microscopy. (B) qRT-PCR analysis of expression of exosomal circMYC after RNase R treatment.

**Figure 2:**
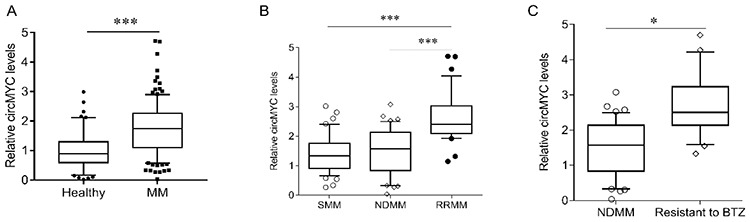
The expression of circMYC in multiple myeloma. (A) The expression of exosomal circMYC in patients with MM and healthy subjects. (B) The expression of exosomal circMYC in SMM, NDMM, and RRMM. (C) The expression of exosomal circMYC in bortezomib-resistant MM and NDMM. MM, Multiple myeloma; NDMM, newly diagnosed MM; RRMM, relapsed/refractory MM; SMM, smoldering multiple myeloma. *p<0.05, ***p<0.001.

In order to study the role of circulating exosomal circRNA in the survival and prognosis of patients with MM, we isolated and purified exosomes, which were confirmed by transmission electron microscopy (Figure 1A). The RNA of the exosomes was extracted. It was found that RNase R treatment could not eliminate the expression of circMYC (Figure 1B). Compared with the healthy population, the expression level of circMYC in the serum exosomes of patients with MM was significantly increased (Figure 2A, p<0.001). Eighty (65.6%) patients had high circMYC levels (Table 1). CircMYC levels in relapsed/refractory MM (RRMM) patients were higher than those of smoldering MM (SMM) and newly diagnosed MM (NDMM) patients (Figure 2B, p<0.001). In addition, during follow-up, 20 patients with BTZ resistance were found to have higher circMYC expression in circulating exosomes than before resistance (Figure 2C, p<0.001).

**Figure 3:**
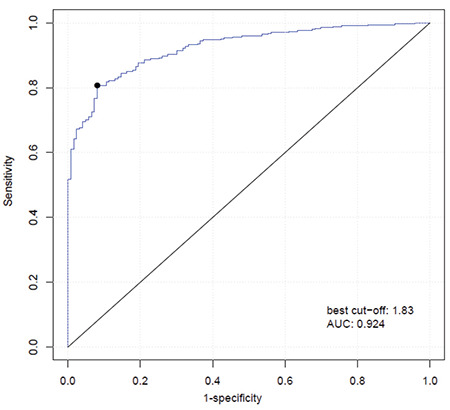
Based on the ROC curve (AUC: 0.924), the best cut-off was 1.83, which was very close to the mean (1.76) of exosomal circMYC in MM patients. We thus used the mean value of circMYC expression as the cutoff value to divide the MM patients into a high circMYC group and a low circMYC group. ROC: Receiver operating characteristic, AUC: area under the curve, MM, multiple myeloma.

We further analyzed the relationship between the expression level of exosomal circMYC and clinical characteristics of patients with MM. Based on the ROC curve (AUC: 0.924; Figure 3), the best cut-off was 1.83, which was very close to the mean (1.76) of exosomal circMYC in MM patients. Therefore, we used the mean value of circMYC expression as the cutoff value to divide the MM patients into a high circMYC group and a low circMYC group. The results showed that, compared with MM patients with low circMYC, the patients with high circMYC had higher percentages of deletion 17p (p=0.0370), t(4;14) (p=0.0127), DSS stage III (p=0.0314), and ISS stage III (p=0.0450) (Table 1). The expression of circMYC in circulating exosomes was not significantly associated with gender, age, deletion 13q, t(14;16), t(14;20), monoclonal immunoglobulin, hypercalcemia, renal insufficiency, anemia, or bone disease (Table 1). Univariate and multivariate Cox regression analysis found that high exosomal circMYC levels were independent predictors of poor prognosis in patients with MM (Tables 2 and 3).

### Association between Expression of Exosomal CircMYC and Relapse and Survival in Patients with MM

MM patients with high exosomal circMYC had higher relapse rates (hazard ratio (HR): 4.05, 95% confidence interval: 2.54-7.37, p=0.0064) and higher mortality rates (HR: 3.67, 95% confidence interval: 1.65-5.58, p=0.0132) (Table 4). Survival analysis also found that MM patients with high exosomal circMYC had lower overall survival (p<0.0001) and progression-free survival (p<0.0001).

## Discussion

There are no obvious clinical symptoms in the early stage of MM, resulting in misdiagnosis. At present, diagnosis is mainly based on bone marrow biopsy to detect the plasma as well as determining whether the patient has anemia, bone pain, hypercalcemia, or kidney dysfunction [21]. Bone marrow puncture smears and fluorescence in situ hybridization are commonly used in clinical examination, but bone marrow aspiration can cause trauma for patients [22]. In addition, during the treatment of MM, most of the patients may develop drug resistance, rendering the original treatment plan ineffective. The above tests have certain limitations in dynamically monitoring the curative effects. However, liquid biopsy is non-invasive, dynamic, reproducible, and low-risk [23]. It can assist in early diagnosis and monitor curative effects (including post-relapse treatment), showing excellent clinical application value [24].

Because circRNAs are conserved and stable in circulation and have high time- and tissue-specific expression patterns, they have potential for clinical application as biomarkers for the diagnosis and prognosis of hematological malignancies [25]. CircRNA is closely related to the malignant transformation, curative effect, and prognosis of hematological malignancies, but the specific molecular mechanism involved in the occurrence and development of the disease remains to be further elucidated [26]. This study found that the expression of circMYC of circulating exosomes in patients with MM was significantly higher than that in healthy people and was significantly correlated with DSS and ISS staging, indicating a poor prognostic outcome. The development of MM generally ranges from monoclonal gammopathy of unknown significance (MGUS) to asymptomatic myeloma, and eventually to symptomatic myeloma. If the patient shows symptoms such as bone pain, anemia, and kidney damage, it indicates that the disease has progressed to stage III. If detected early in the MGUS and SMM stages, patients can receive timely and effective intervention. Iaccino et al. [7] reported that idiotype-specific binding peptides can quickly and simply identify exosomes released by MM cells, and they also found that MM-derived exosomes can be used to determine the progress of MM early compared with serum paraprotein IgG2b. Therefore, circulating exosomes may be useful tools for early detection in patients with MM.

Acquired resistance is a major obstacle to tumor chemotherapy. The communication between tumor cells and the microenvironment plays an important role in malignant tumor transformation [27]. Studies have found that exosomes can promote tumor cell proliferation, metastasis, and drug resistance in the tumor microenvironment. Exosomes contain miRNAs and circRNAs that can be transferred to recipient cells to regulate protein expression and signal pathway transduction. Recently, exosomes have been shown to be closely related to tumor development and metastasis as well as drug resistance [28]. It was reported that cisplatin can lead to increased secretion of exosomes in lung cancer cells, and the expression levels of certain miRNAs (miR-21, miR-133b) in exosomes are related to cisplatin resistance [29]. A recent study found that circular RNA Cdr1as was downregulated in serum exosomes from cisplatin-resistant patients with ovarian cancer, and overexpression of Cdr1as increased the cisplatin-induced cell apoptosis in ovarian cancer cells [30]. It was also reported that circ_0000190 was downregulated in both bone marrow tissue and peripheral blood from patients with MM, and overexpression of circ_0000190 inhibited MM cell growth in vitro and in vivo through targeting the miR-767-5p/MAPK4 axis [31]. The clinical application of BTZ has improved the remission rate and survival rate of patients with MM. Bandari et al. [32] reported that the exosomes derived from myeloma cells were increased significantly by BTZ. Bone marrow mesenchymal stem cells (BMSCs) and MM cells regulate each other through exosomes, and BMSCs making MM cells evade anti-myeloma treatment is one of the factors leading to drug resistance [33]. BTZ induces MM cell death by regulating bcl-2, caspase-9, and caspase-3, while BMSC-derived exosomes inhibit the function of BTZ, leading MM cells to become resistant [33]. In this study, we found that the expression of circMYC in circulating exosomes in drug-resistant patients was significantly higher than that in non-resistant patients, suggesting that the expression of circMYC is associated with drug resistance in MM patients. A previous study found that circMYC was significantly associated with drug response to multiple drugs, including belinostat (an HDAC inhibitor) and cetuximab, in ways that are beyond the known mechanisms, including genomic variation and transcriptomic variation [16]. Therefore, these findings provide new ideas for the treatment of MM by targeting exosomes, which has the advantages of stability, non-toxicity, and strong delivery through blocking the communication between tumor cells and the tumor microenvironment.

### Study Limitations

There are some shortcomings in our study. For example, the scale of the cohort was small and the patients’ information was analyzed based on retrospective data. In addition, this study lacks functional investigation in vivo and in vitro to explore the potential role and underlying mechanisms of circMYC in MM. Cell biological experiments and prospective studies in humans should be performed in the future to confirm the conclusions of this study.

## Conclusion

This study has demonstrated that circulating exosomal circMYC is upregulated in patients with MM and is associated with BTZ response and relapse, which indicates that exosome circMYC has great potential as a biomarker for the diagnosis and prognosis of MM. CircRNA is expected to be applied for the minimally invasive diagnosis and prognosis of hematological malignancies in the near future, and it may become a potential therapeutic target.

## Figures and Tables

**Table 1:**
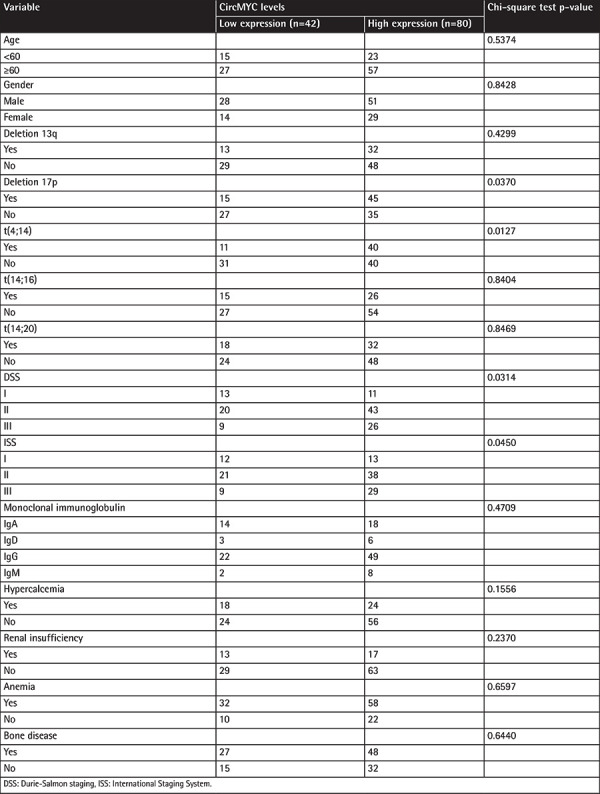
Clinical association between circMYC levels and clinicopathological variables of patients with multiple myeloma.

**Table 2:**
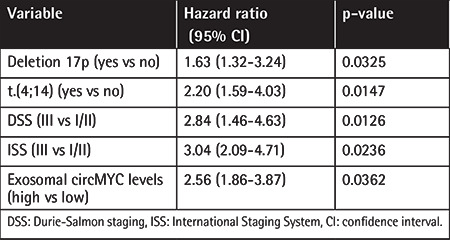
Univariate analysis of prognostic factors of MM.

**Table 3:**
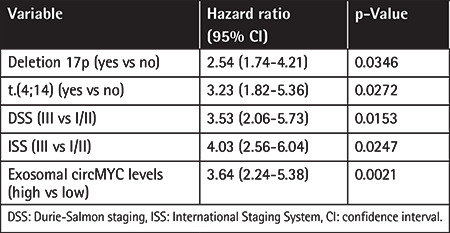
Multivariate analysis of independent prognostic factors of MM.

**Table 4:**
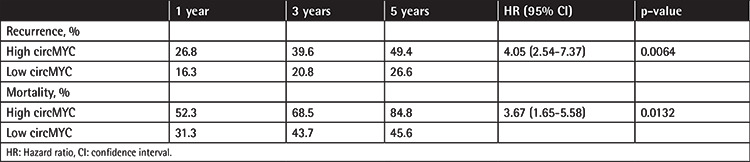
Association of recurrence and mortality and circMYC expression.

**Figure 4:**
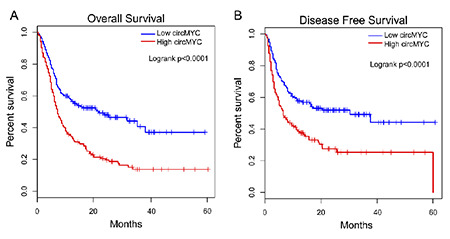
High exosomal circMYC is associated with worse survival rates in patients with MM. (A) The overall survival rate was evaluated by Kaplan-Meier curve. (B) The disease-free survival rate was evaluated by Kaplan-Meier curve.
